# Improving the characterization of dissolved organic carbon in cloud water: Amino acids and their impact on the oxidant capacity

**DOI:** 10.1038/srep37420

**Published:** 2016-11-23

**Authors:** Angelica Bianco, Guillaume Voyard, Laurent Deguillaume, Gilles Mailhot, Marcello Brigante

**Affiliations:** 1 Université Clermont Auvergne, Université Blaise Pascal, Institut de Chimie de Clermont-Ferrand, BP 10448, F-63000 Clermont-Ferrand, France; 2 CNRS, UMR 6296, ICCF, F-63171 Aubiere, France; 3 Université Clermont Auvergne, Université Blaise Pascal, OPGC, Laboratoire de Météorologie Physique, BP 10448, F-63000 Clermont-Ferrand, France; 4 CNRS, UMR 6016, LaMP/OPGC, BP80026, F-63177 Aubiere, France

## Abstract

Improving our understanding of cloud chemistry depends on achieving better chemical characterization (90% of the organic carbon [OC] fraction remains uncharacterized) and, consequently, assessing the reactivity of this complex system. In this manuscript, we report for the first time the concentrations of 16 amino acids (AAs) in 25 cloud water samples. The concentrations of individual AAs ranged from a few nM up to ~2.0 μM, and the average contribution of AAs corresponded to 9.1% (4.4 to 21.6%) of the dissolved OC (DOC) concentration. Considering their occurrence and concentrations, AAs were expected to represent an important hydroxyl radical (HO^•^) sink in aqueous cloud samples. In this work, we estimated that approximately 17% (from 7 to 36%) of the hydroxyl radical-scavenging ability of the DOC could be attributed to the presence of AAs, whereas comparing the AAs suggested that an average of 51% (from 22 to 80%) of their reactivity with HO^•^ could account for the presence of tryptophan. These results clearly demonstrate that the occurrence and reactivity of AAs must be considered to better estimate the chemical composition and oxidant capacity of the cloud aqueous phase.

The organic carbon (OC) fraction of aerosol accounts for a large portion of air particulate matter and exhibits many complex molecular structures^1^. This organic matter originates from a wide variety of natural and anthropogenic sources; additionally, it undergoes chemical transformations during its atmospheric lifetime^2^ and strongly influences the climate and health effects of atmospheric aerosols^3^. In the OC fraction of aerosols, water-soluble OC (WSOC) is especially interesting because it contributes to particles’ hygroscopic properties and participates in aqueous cloud chemistry^4^. Some WSOC components are emitted into the air as primary particles (*e.g.,* through biomass burning), and others are produced by reactions in the gas and aqueous phases^5^,^6^. One major concern is related to the effects of WSOC on the surface tension of particles and their ability to be activated into cloud droplets^7^. However, a large fraction of WSOC remains uncharacterized, and substantial work has focused on quantifying short-chain mono and di-carboxylic acids (CAs) and carbonyl and dicarbonyl compunds^8^,^9^.

Soluble organic matter comprises small volatile molecules associated with larger multifunctional structures containing a substantial fraction of hetero-atoms (*e.g.,* N, S, and O), such as HUmic LIke Substances (HULIS), and polyols, such as sugars, phenols, and polyconjugated species^10^,^11^. Multiple functionalities, including hydroxyl, carboxyl and carbonyl groups, are present in these molecules. Field measurements have revealed that a large part of this WSOC remains not characterized despite the application of a variety of analytical approaches.

Among these compounds, the presence of amino acids (AAs) belonging to proteinaceous matter was highlighted in rain, fog waters and aerosols^12^,^13^. Their effect on the surface tension properties of aerosols and their role as ice nuclei were studied, revealing that AAs could affect the atmospheric water cycle^14^,^15^,^16^,^17^. Proteinaceous matter is injected into the atmosphere by the production of sea salt aerosol when air bubbles burst at the ocean surface. Proteinaceous matter can also be emitted in the atmosphere by the activity of bacteria and phytoplankton or from the injection of organic film into the air at the sea surface^18^,^19^,^20^. Over continental areas, proteinaceous matter is njected into the atmosphere as plant debris, pollen, and algae, and this biological fraction has been frequently detected on aerosol particles^21^. The first oxidation of the proteinaceous matter could occur on the aerosol particle, for example, involving methionine and tryptophan, which are rapidly oxidized when exposed to ozone and sunlight^22^.

One important difference between the free and combined forms of AAs must be noted when analysing environmental samples (*e.g*., rain, fog, clouds, and aerosols)^20^,^23^. The analysis of aerosol and fog samples revealed that AAs were mostly present as dissolved combined AAs (DCAAs), such as proteins, peptides and HULIS^20^,^24^,^25^. One crucial question relates to how proteins and peptides are converted into free AAs (dissolved free AAs [DFAAs]). Milne and Zika proposed different hypotheses about aerosol samples, excluding thermal hydrolysis but suggesting that enzymatic hydrolysis could be not-negligible^26^. This hypothesis was corroborated by recent works revealing the presence of living microorganisms and impact on the oxidant capacity and organic speciation in cloud water^27^,^28^. The direct photolysis of peptides and photosensitized reaction pathways could be responsible for their fragmentation under actinic radiation^29^. Thus, in environmental samples, both forms of AAs could be detected, but the concentration of DFAAs (uncombined forms) is expected to be lower because this is the preferred form for the uptake of nitrogen compounds by microorganisms^20^. Recent works have shown that AAs dissolved in cloud water could be transformed by solar radiation and reaction with oxidative species (*i.e.,* hydroxyl radicals), leading to the production of different functionalized and oxidized products and short-chain CAs^30^,^31^.

In this manuscript, we report the AA concentrations in 25 cloud water samples collected at puy de Dôme Mountain (France) during 11 cloud events. Chromatographic separation coupled with chemical complexation and fluorescence detection was used to determine the concentration of DFAAs. To the best of our knowledge, this study reports, for the first time, the concentrations of free AAs in cloud water. Comparing the reactivities of the AAs with those of naturally occurring CAs and dissolved OC (DOC) suggested that the presence and reactivity of AAs should be considered in the future to better assess the effect of cloud aqueous-phase chemistry on the organic matter transformation mediated by the hydroxyl radical (HO^•^).

## Results and Discussions

### Quantification of AAs

The concentrations of 16 AAs were determined in 25 individual samples from 11 cloud events collected during two campaigns in March/April and November 2014 at the top of puy de Dôme mountain (1465 m a.s.l.) in France. The cloud droplet sampling was performed using a one-stage cloud droplet impactor, as previously described^8^. The sampling times ranged from 120 to 180 min depending on the liquid water content, which reflects the mass of water in a cloud in a specified amount of dry air and was in the range of 0.1–0.2 g m^−3^. Single-AA concentrations ranged from 5 nM (corresponding to the analytical detection limit) to ~2.0 μM (Supplementary Table S1).

**Figure 1:**
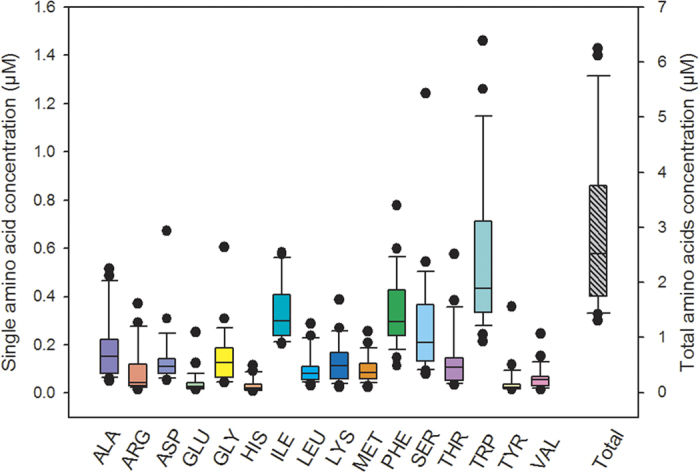
Distribution of each AA in the cloud samples. The bottom and top lines of the box correspond to the 25^th^ and 75^th^ percentiles, respectively. The middle line represents the median. The ends of the whiskers are the 10^th^ and 90^th^ percentiles, and the filled circle is an outlier. The y-right scale shows the total AA concentration.

Figure 1 illustrates the concentrations of each AA in the cloud samples, showing that the average concentrations of ILE, PHE, SER and TRP exceeded 0.25 μM (see Table 1 for the AA abbreviations and relative concentrations). The median values of the single-AA concentrations were lower than 0.5 μM, and concentrations of ILE and TRP were the highest, with an average value of 0.3 and 0.4 μM respectively. The total AA concentration, defined as the median value of the sum of the AA concentration in each sample, ranged from 1 to 4 μM. The average concentrations of AA in cloud water samples were lower than those measured in fog water by Zhang *et al*.^13^ (2.6–99 μM, mean ± 1σ = 20 ± 27 μM) and in dew water samples in Germany by Scheller^32^ (0.5–110 μM, mean = 22.5 μM). The concentrations found here were relatively similar to those reported in rainwater by Gorzelska *et al*.^33^ (≈ 0.4 μM) and Mopper and Zika^34^ (6.5 μM). Fog and dew water were expected to be more concentrated than cloud water because they are influenced relatively strongly by local sources and lead to less dilution of particle compared to the cloud droplets. The sampling procedure may also have contributed to this difference because of the potential evaporation of the liquid sample. The high TRP concentration cannot be directly correlated to the hydrolysis of proteinaceous matter. However, TRP was recently demonstrated to be strongly correlated with the formation of high-molecular-weight compounds with spectroscopic characteristics similar to those of HULIS, which can also be considered as a potential TRP reservoir^30^,^35^. Muller *et al*.^36^ evaluated TRYptophan LIke Substances (TRYLIS) in rain using fluorescence emission. These authors found that the TRP concentration was higher in samples from marine origins, suggesting that the presence of TRYLIS promoted the development of ice-precipitation at ‘warmer’ temperatures. Nevertheless, the detection of this compound in the fluorescence matrix was not suitable for the discrimination of free and combined TRP.

**Figure 2:**
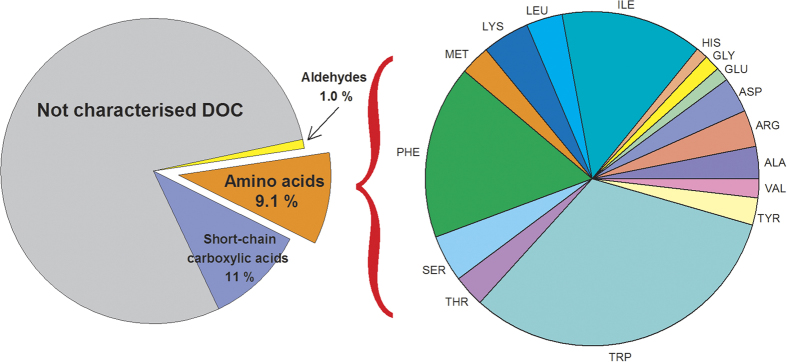
Pie plot showing the distributions of AAs, aldehydes and the most relevant short-chain CAs (formate, acetate, oxalate, malonate and succinate) relative to the total DOC in cloud water samples. The percentages were calculated based on the average concentrations of individual AAs, aldehydes, CAs and DOC in mg C L^−1^. The second plot shows the distribution of a single AA relative to the total distribution.

The proportion of AAs to the DOC ranged from 4.4 to 21.6% of the total carbon (with an average value of 9.1%) in 25 samples, corresponding to an average estimated concentration of 211 ± 19 μg C L^−1^ (see Table 1 for the average AA concentrations). Deguillaume *et al*. suggested that the characterization of the organic matter in cloud water remains incomplete because approximately 11% and 1% of the DOC is represented by short-chain CAs (formate, acetate, oxalate, malonate and succinate) and aldehydes, respectively^8^. In Fig. 2 the average contributions of CAs, aldehydes and AAs to the DOC are presented, and the second pie plot reports the proportion of each AA.

### Reactivity with HO^•^: Competition among AAs, CAs and DOC

**Figure 3:**
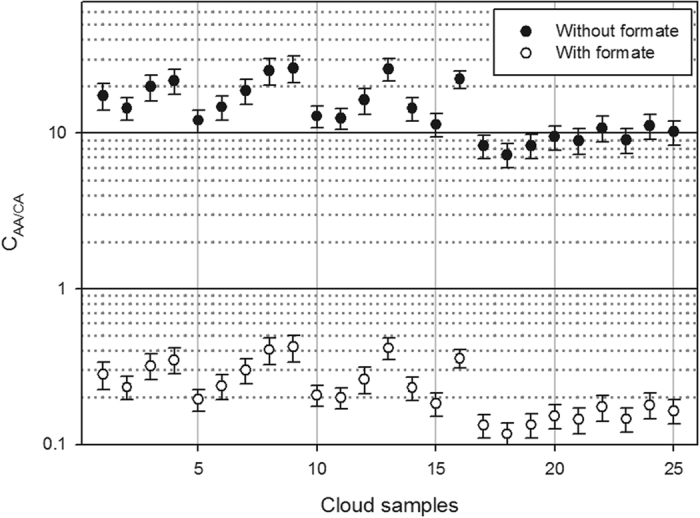
Ratio of the scavenging rate constants of HO^•^ (expressed in s^−1^) with AAs and short-chain CAs (C_AA/CA_) without (filled circles) and with (empty circles) formate (calculated using equation 1). The error bars were determined by considering the uncertainties associated with AA quantification in each sample.

The reactivity of AAs with hydroxyl radicals can be calculated and compared with those of most relevant short-chain CAs (formate, acetate, oxalate, malonate and succinate) always measured in the cloud aqueous phase^8^,^37^,^38^. This competition (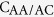) can be evaluated by considering the ratios between the scavenging rate constants of HO^•^ by AA (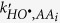) and CA (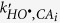) using Equation (1):


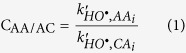


where 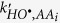
and 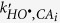
are determined as the product of the second-order rate constant of HO^•^ with single AAs and CAs 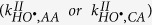 and their concentrations, respectively. Based on the average CA concentration in the aqueous cloud phase reported by Deguillaume *et al*.^8^ (Supplementary Table S2) and the AA concentrations determined in each sample, 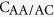 can be calculated by considering the contribution (or not) of formate to the CAs and a pH of 5.0 (see Supplementary Tables S3 and S4 for the pKa values and HO^•^ reactivities of each species and the DOC concentrations in each cloud sample, respectively). As shown in Fig. 3, for the comparison without formate, the AA contribution was between ~7 and 26 times larger than that of the CAs. Interestingly, for all samples, the scavenging abilities (corresponding to the pseudo-first order decay constant between considered species and hydroxyl radical) of the AAs were at least one order of magnitude higher than those of the short-chain CAs. In terms of the proportion of formate among the short-chain CAs, the 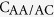 value ranged from 0.12 to 0.42. These findings seem reasonable given the high reactivity of formate with HO^•^ 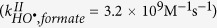. Nevertheless, at lower pH values, the contribution of formate was expected to be relatively small; indeed, at pH 4.0, approximately 37% of formate exists as its acidic form (*i.e*., formic acid, pKa = 3.76), which is less reactive towards HO^•^ 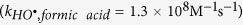.

**Figure 4:**
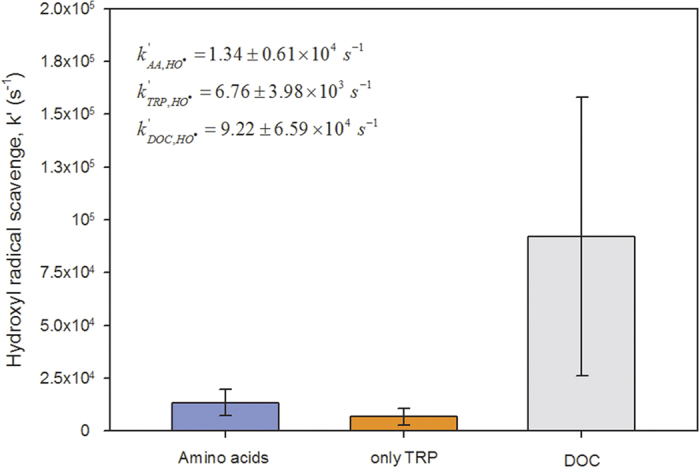
HO^•^ scavenging rates (k′) of AAs, TRP and DOC. These values were determined based on the average values of 
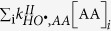
, 
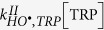
 and 
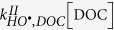
 using the concentrations and second-order rate constants reported in Tables S1 and S3. The 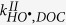 value was taken from Arakaki *et al*.^39^.

Figure 4 shows the contributions of all AAs, TRP and DOC to the scavenging rate constant of the hydroxyl radical in terms of their average concentrations. Up to 51% of the total reactivity of HO^•^, which is expressed as 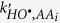, was attributable to TRP (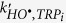
~ 6.76 ± 3.98 × 10^3^ s^−1^) among all AAs (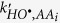 ~1.28 ± 0.51 × 10^4^ s^−1^). Interestingly the concentration of TRP was expected to be lower than those of other AAs because of its high reactivity with HO^•^ 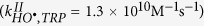, suggesting the presence of a possible source of TRP in cloud water^28^. Moreover, as recently reported, the oxidation of TRP in cloud waters led to the formation of formate and acetate in 20% yields, with other nitrogen derivatives also likely produced (up to 60% of the uncharacterized carbon)^30^. These findings demonstrated that TRP oxidation could be responsible for a large fraction of the HO^•^ scavenged in natural samples.

Moreover, the relative contributions of AAs to the DOC’s reactivity with HO^•^ can be estimated using Equation (2):


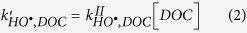


where 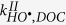 and 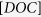 are the second-order rate constant between HO^•^ and DOC estimated by Arakaki *et al*.^39^ to be 3.8 × 10^8^ L (mol C)^−1^ s^−1^) and the concentration of DOC, which varied from 1.0 mg C L^−1^ to 8.6 mg C L^−1^, with an average of 2.91 mg C L^−1^ (Supplementary Table S4). In our samples, 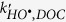 was in the range 0.32–2.72 × 10^5^ s^−1^. This value is similar to those previously determined by Arakaki and Faust, who reported a HO^•^ scavenging rate range of 0.95–4.2 × 10^5^ s^−1^ in 7 cloud water samples collected from Whiteface Mountain^40^. From the quantification of hydroxyl radicals reactivity toward AAs in each cloud sample (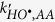) determined considering the second order rate constant between AA and HO^•^ (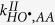) expressed in L (mol C)^−1^ s^−1^ and AA concentration ([AA]) in molC L^−1^ (see TableS3) we can argue that 7% to 36% of the total hydroxyl radicals scavenged by DOC were attributable to the presence of AAs (see Supplementary Table S5 for the calculations performed for each cloud sample).

Finally, AAs constituted a large fraction of the OC in cloud waters and play an important role in the scavenging of generated hydroxyl radicals. The origins of these AAs remain unknown, and their concentrations could not be explained by considering only particle dissolution during cloud droplet formation. This observation suggests the presence of *in-situ* sources (*i.e.,* macromolecule oxidation or microorganism metabolism) that should be investigated in the future. However, our findings implied that the oxidant capacity of this medium (mainly related to HO^•^ reactivity) was strongly influenced by the presence of AAs, which can both act as sink but also modify the hydroxyl radical photogeneration processes *via* iron (Fe^2+^/Fe^3+^) complexation in cloud water. In fact, the complexation of ferrous and ferric ions with amino acids in water has been reported. Stability constants of Fe-amino acids complexes result in some case (*i.e.*, for Glycine, Tryptophan, Valine) to be higher that the value reported for Fe-carboxylate complexes^41^,^42^,^43^. We demonstrated that the presence of AAs in cloud waters must be accounted for to facilitate predicting the cloud water oxidizing capacity using multiphase chemistry models.

### Experimental Materials and methods

#### AA analysis

**Figure 5:**
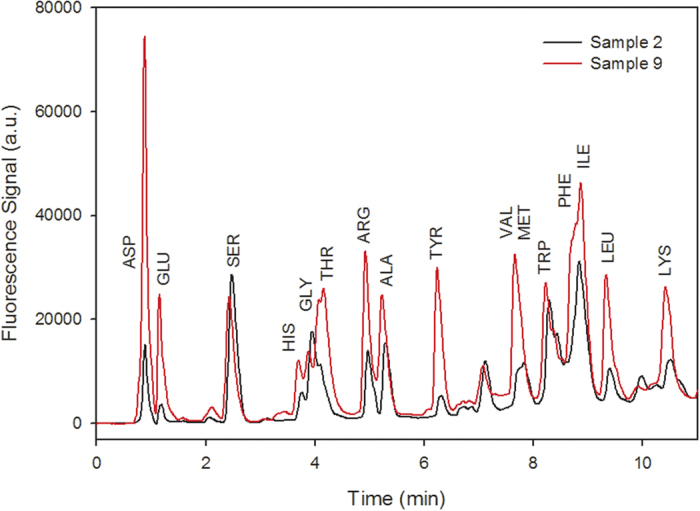
Example HPLC chromatograms obtained from samples 2 and 9.

Cloud water samples were filtered immediately after collection using a 0.45-μm polytetrafluoroethylene (PTFE) filter to eliminate any microorganisms or particles. The solutions were stored at 255 K in the dark before analysis. The derivatization adopted was based on a previous method described by Ishida *et al*.^44^ and adapted to the cloud water analysis of primary AAs. Essentially, the -NH_2_ groups of primary AAs react with *o*-phthalaldehyde (OPA) to form a fluorescent derivative in the presence of a thiol group (here, mercaptopropionic acid [MPA]), which acts as a reaction catalyst. Secondary AAs were previously reported to be unreactive with OPA. After reacting with OPA, the cloud water samples were analysed by high-performance liquid chromatography (HPLC; Shimadzu Nexera equipped with an autosampler/pretreatment unit (SIL-30AC). For this purpose, 30 μL of MPA (7.7 mM in 0.1 M borate buffer), 15 μL of OPA (15.0 mM in 0.1 M borate buffer) and 5 μL of sample were mixed in a vial, and after 35 min, which is the length of time required for complete complexation, 20 μL of the solution was injected. Chemicals were purchased from Sigma-Aldrich and all solutions were prepared using MilliQ water (≥18.2 MΩ cm). The derivatized compounds were separated with an HPLC column (Shimadzu Shim-pack XR-ODS; 3.0 × 100 mm, Ø 2.2 μm porous particles) and eluted at a flow rate of 0.7 mL min^−1^ using a gradient program with two eluents: eluent A (10 mM phosphate buffer, pH 6.8) and eluent B (acetonitrile, methanol and water 45:45:10 v/v/v). The gradient elution was as follows: initially, 10% of (B); a linear gradient to 75% (B) within 15 min; a faster increase of (B) to 100% in 1 min (corresponding to 16 min of elution); and constant (B) for an additional 5 min. A fluorescence detector (RF-20A XS) was used to detect AA-OPA derivatives at an excitation wavelength (λ_ex_)/emission wavelength (λ_em_) 350/450 nm (Fig. 5 shows the HPLC chromatograms of samples 2 and 9).

The standard solution was prepared by diluting the AA-S-18 AA standard solution (Sigma Aldrich) in 0.1-M HCl (Supplementary Figure S1). Cloud water samples were analysed without further pre-treatment.

Each analysis was evaluated and monitored in terms of the reproducibility of the peak retention times and peak heights and the linearity of the calibration curve. The method’s limit of detection (LOD) was estimated to be on the order of 5 nM with the injection of 20 μL of sample. Blank experiments (MilliQ water subjected to the entire derivatization process) were performed and confirmed that the AA concentration in water was always lower than the LOD.

In this work, only the DFAA concentration was quantified. The samples were subjected to acidic hydrolysis according to the method described by Fountoulakis *et al*.^45^ (1.5 mL of sample combined with 6 M HCl in an ampoule and then heated at 100 °C for 60 min). However, both the combined and free AAs were hydrolysed, and the concentration was below the LOD. No significant differences in the AA concentration were found using filtered *vs.* unfiltered samples or before and after freezing.

Calibration curves (single amino acid area *vs.* injected concentration) were performed using a cloud water matrix in order to consider the interferences on the signal in amino acids quantification (Figure S2).

## Additional Information

**How to cite this article**: Bianco, A. *et al*. Improving the characterization of dissolved organic carbon in cloud water: Amino acids and their impact on the oxidant capacity. *Sci. Rep.* **6**, 37420; doi: 10.1038/srep37420 (2016).

**Publisher's note:** Springer Nature remains neutral with regard to jurisdictional claims in published maps and institutional affiliations.

## Supplementary Material

Supplementary Information

## Figures and Tables

**Table 1:** Average single-AA concentration (nM) and corresponding average carbon concentration expressed in μg C L^−1^.

Compound	Abbreviation	Molecular weight (g mol^−1^)	Average concentration (nM)	Carbon average concentration (μg C L^−1^)
Alanine	ALA	89.0	188 ± 133	6.8 ± 4.8
Arginine	ARG	174.2	89 ± 95	6.4 ± 6.8
Aspartate	ASP	133.1	139 ± 124	6.7 ± 5.9
Glutamate	GLU	147.1	42 ± 49	2.5 ± 2.9
Glycine	GLY	75.1	151 ± 117	3.6 ± 2.8
Histidine	HIS	155.2	33 ± 28	2.4 ± 2.0
Isoleucine	ILE	131.2	548 ± 532	24.0 ± 8.3
Leucine	LEU	131.2	102 ± 66	7.4 ± 4.8
Lysine	LYS	146.2	125 ± 87	9.0 ± 6.2
Methionine	MET	149.2	100 ± 56	6.0 ± 3.4
Phenylalanine	PHE	165.2	337 ± 152	36.4 ± 16.4
Serine	SER	105.1	281 ± 238	10.1 ± 8.6
Threonine	THR	119.1	133 ± 125	6.4 ± 6.0
Tryptophan	TRP	204.2	563 ± 332	74.3 ± 43.8
Tyrosine	TYR	181.2	45 ± 69	4.9 ± 7.4
Valine	VAL	117.2	61 ± 50	3.7 ± 3.0
Total			2938 ± 167	211 ± 19

Values are presented with the standard deviation determined by analysing 25 samples.
