# Supplementary material for: Improving the characterization of dissolved organic carbon in cloud water: Amino acids and their impact on the oxidant capacity
Source: Sci Rep. 2016 Nov 23;6:37420. doi: 10.1038/srep37420 (PMC5120292; doi:10.1038/srep37420)
Supplement: Supplementary Information [file srep37420-s1.doc]

***Supplementary Materials***

**Improving the characterization of dissolved organic carbon in cloud water: Amino acids and their impact on the oxidant capacity**

**Angelica Bianco^a,b^, Guillaume Voyard^a,b^, Laurent Deguillaume^c,d^, Gilles Mailhot^a,b^, Marcello Brigante^a,b*^**

*^a^ Université Clermont Auvergne, Université Blaise Pascal, Institut de Chimie de Clermont-Ferrand, BP 10448, F-63000 CLERMONT-FERRAND, FRANCE*

*^b^ CNRS, UMR 6296, ICCF, F-63171 AUBIERE, FRANCE*

*^c^* *Université Clermont Auvergne, Université Blaise Pascal, OPGC, Laboratoire de Météorologie Physique, BP 10448, F-63000 CLERMONT-FERRAND, FRANCE*

*^d^* *CNRS, UMR 6016, LaMP/OPGC, BP80026, F-63177 AUBIERE, FRANCE*

* Corresponding author Marcello Brigante: University Blaise Pascal, Institute of Chemistry of Clermont-Ferrand, avenue des Landais 63171 Aubière, France; Phone +33 0473405514 e-mail: marcello.brigante@univ-bpclermont.fr

| **Cloud event** | **Sample** | **Sampling**  **period and time**  **(dd/mm/time)** | **Concentrations (nM)** | | | | | | | | | | | | | | | |
| --- | --- | --- | --- | --- | --- | --- | --- | --- | --- | --- | --- | --- | --- | --- | --- | --- | --- | --- |
|  |  |  | **ALA** | **ARG** | **ASP** | **GLU** | **GLY** | **HIS** | **ILE** | **LEU** | **LYS** | **MET** | **PHE** | **SER** | **THR** | **TRP** | **TYR** | **VAL** |
| I | 1 | 22/03 7am-11pm | 295±9 | 24±5 | 172±9 | 33±2 | 194±14 | 17±5 | 413±47 | 94±11 | 386±157 | 256±18 | 329±42 | 545±43 | 123±5 | 487±102 | 42±5 | 44±5 |
| II | 2 | 25/03 11am-1pm | 221±7 | 47±5 | 147±8 | 37±5 | 140±10 | 16±5 | 370±42 | 80±9 | 46±19 | 106±7 | 425±54 | 392±31 | 111±5 | 568±118 | 25±5 | 57±5 |
| III | 3 | 26/03 8am-9am | 140±5 | 29±5 | 83±4 | 22±5 | 126±9 | 31±5 | ND | 111±13 | 113±46 | 81±6 | 543±69 | 205±16 | 109±5 | 943±197 | 17±5 | 61±5 |
| IV | 4 | 26/03 9am-11am | 514±17 | 21±5 | 109±6 | 55±5 | 176±12 | 35±5 | ND | 102±11 | 86±35 | 62±5 | 598±76 | 227±18 | 150±5 | 1072±224 | 20±5 | 67±5 |
|  | 5 | 04/04 8pm-10pm | 189±6 | 45±5 | 160±9 | 55±5 | 109±8 | 17±5 | 417±47 | 109±12 | 25±10 | 91±6 | 289±31 | 242±19 | 145±5 | 476±99 | 29±5 | 56±5 |
| V | 6 | 04/04 10pm-12pm | 152±5 | 28±5 | 88±5 | 26±5 | 104±7 | 20±5 | 510±57 | 99±11 | 56±23 | 29±5 | 390±49 | 211±16 | 123±5 | 640±134 | 17±5 | 31±5 |
|  | 7 | 04-05/04 1pm-4am | 90±5 | 31±5 | 53±5 | 21±5 | 209±15 | 92±6 | 331±37 | 41±5 | 44±18 | 69±5 | 270±34 | 172±13 | 90±5 | 986±206 | 16±5 | 58±5 |
|  | 8 | 05/04 4am-7am | 138±5 | 32±5 | 98±5 | 25±5 | 182±13 | 30±5 | ND | 166±19 | 102±41 | 77±5 | 297±38 | 182±14 | 52±5 | 1460±305 | 24±5 | 73±5 |
|  | 9 | 05/04 7am-10am | 226±7 | 17±5 | 139±7 | 39±5 | 309±22 | 34±5 | ND | 222±25 | 215±88 | 171±12 | 518±66 | 372±29 | 336±10 | 1260±263 | 19±5 | 63±5 |
| VI | 10 | 04/11 11am-1pm | 486±16 | 267±10 | 209±11 | 124±9 | 229±16 | 57±5 | 205±23 | 171±19 | 204±83 | 165±11 | 352±45 | 475±37 | 147±5 | 214±45 | 78±5 | 154±6 |
| VII | 11 | 05/11 4am-6pm | 207±7 | 141±5 | 134±7 | 36±5 | 243±17 | 55±5 | 351±39 | 111±13 | 67±27 | 116±8 | 316±40 | 468±36 | 138±5 | 377±79 | 54±5 | 92±5 |
|  | 12 | 05/11 7am-9am | 209±7 | 131±5 | 131±7 | 53±5 | 147±10 | 37±5 | 404±45 | 92±10 | 270±110 | 94±7 | 452±57 | 250±19 | 61±5 | 506±106 | 33±5 | 62±5 |
|  | 13 | 05/11 9am-12am | 453±15 | 113±5 | 308±17 | 45±5 | 604±42 | 83±5 | 584±66 | 238±27 | 196±80 | 209±15 | 777±98 | 1242±97 | 385±11 | 782±163 | 118±5 | 113±5 |
| VIII | 14 | 12/11 5pm-7pm | 74±5 | 293±11 | 107±6 | 27±5 | 61±5 | 39±5 | 300±34 | 67±8 | 137±56 | 128±9 | 296±38 | 102±8 | 59±5 | 486±101 | 41±5 | 37±5 |
|  | 15 | 12/117pm-9pm | 50±5 | 180±7 | 75±5 | 19±5 | 46±5 | 26±5 | 287±32 | 63±7 | 80±33 | 99±7 | 272±34 | 79±6 | 36±5 | 402±84 | 21±5 | 33±5 |
|  | 16 | 12/11 9pm-12pm | 332±11 | 372±14 | 673±36 | 252±18 | 146±10 | 116±8 | 575±65 | 289±33 | 251±102 | 169±12 | 433±55 | 362±28 | 576±17 | 347±72 | 358±11 | 247±10 |
| IX | 17 | 14/11 5pm-7pm | 99±5 | 107±5 | 95±5 | 21±5 | 102±7 | 19±5 | 207±23 | 57±6 | 58±24 | 53±5 | 148±19 | 163±13 | 55±5 | 321±67 | 26±5 | 24±5 |
| X | 18 | 17/11 4pm-6pm | 68±5 | 47±5 | 69±5 | 16±5 | 53±5 | 12±5 | 327±37 | 31±4 | 33±13 | 24±5 | 114±14 | 107±8 | 52±5 | 314±66 | 19±5 | 18±5 |
|  | 19 | 17/11 7pm-9pm | 68±5 | 76±5 | 82±5 | 18±5 | 57±5 | 18±5 | 241±27 | 52±6 | 114±46 | 84±6 | 204±26 | 109±9 | 42±5 | 244±51 | 23±5 | 42±5 |
|  | 20 | 17/11 9pm-12pm | 61±5 | 42±5 | 56±5 | 14±5 | 48±5 | 14±5 | 230±26 | 60±7 | 61±25 | 51±5 | 253±32 | 94±7 | 47±5 | 375±78 | 21±5 | 14±5 |
|  | 21 | 18/11 6am-8am | 119±5 | 36±5 | 81±5 | 22±5 | 84±6 | 12±5 | 240±27 | 59±7 | 131±53 | 57±5 | 201±25 | 206±16 | 107±5 | 305±64 | 28±5 | 31±5 |
|  | 22 | 18/11 5pm-7pm | 155±5 | 39±5 | 123±7 | 20±5 | 124±9 | 13±5 | 261±29 | 59±7 | 142±58 | 62±5 | 227±29 | 282±22 | 116±5 | 404±84 | 28±5 | 29±5 |
|  | 23 | 18/11 7pm-9pm | 75±5 | 30±5 | 66±5 | 21±5 | 45±5 | 9±5 | 237±27 | 51±6 | 70±28 | 52±5 | 238±30 | 102±8 | 35±5 | 356±74 | 19±5 | 21±5 |
| XI | 24 | 18/11 9pm-12pm | 105±5 | 35± | 111±6 | 21±5 | 69±5 | 10±5 | 271±30 | 57±6 | 119±48 | 77±5 | 242±31 | 159±12 | 67±2 | 435±91 | 26±5 | 31±5 |
|  | 25 | 19/11 12pm-2am | 185±6 | 52±5 | 115±6 | 29±5 | 156±11 | 18±5 | 242±27 | 72±8 | 123±50 | 106±7 | 245±31 | 267±21 | 161±5 | 323±67 | 30±5 | 70±5 |

Table S1: Amino acids concentrations in each cloud water sample. Number of cloud events is given in Roman. Errors are given considering 2 separates injections. ND: not determined

| **Compounds** | **Concentrations (µM)** |
| --- | --- |
|  |  |
| Formate | 17.5 |
| Acetate | 6.5 |
| Oxalate | 3.0 |
| Malonate | 0.6 |
| Succinate | 0.4 |

Table S2: Concentrations of carboxylic acids considered for the calculation of competition with amino acids. Data are taken from the average concentrations reported by Deguillaume et al^1^.

| **Compounds** | **pKa** | $k_{HO^{\bullet}} (M^{-1}s^{-1})$ | $k_{HO^{\bullet}} (L molC^{-1}s^{-1})$ |
| --- | --- | --- | --- |
| ALA | 2.3 | 7.7 × 10^9^ | 1.90 × 10^10^ |
| ARG | 2.2 | 3.5 × 10^9^ | 8.47 × 10^9^ |
| ASP | 2.2 | 7.5 × 10^7^ | 2.08 × 10^8^ |
| GLU | 2.2 | 2.3 × 10^8^ | 5.64 × 10^8^ |
| GLY | 2.3 | 1.7 × 10^7^ | 5.32 × 10^7^ |
| HIS | 1.8 | 5.0 × 10^9^ | 1.08 × 10^10^ |
| ILE | 2.4 | 1.8 × 10^9^ | 3.28 × 10^9^ |
| LEU | 2.4 | 1.7 × 10^9^ | 3.10 × 10^9^ |
| LYS | 2.2 | 3.5 × 10^8^ | 7.11 × 10^8^ |
| MET | 2.3 | 8.5 × 10^9^ | 2.11 × 10^10^ |
| PHE | 1.8 | 6.9 × 10^9^ | 1.06 × 10^10^ |
| SER | 2.2 | 3.2 × 10^8^ | 9.34 × 10^8^ |
| THR | 2.6 | 5.1 × 10^8^ | 1.27 × 10^9^ |
| TRP | 2.4 | 1.3 × 10^10^ | 2.01 × 10^10^ |
| TYR | 2.2 | 1.3 × 10^10^ | 2.18 × 10^10^ |
| VAL | 2.3 | 8.5 × 10^8^ | 1.66 × 10^9^ |
| Formate  Acetate  Oxalate succinate  Malonate | 3.76  4.75  1.25–4.23  4.23–5.64  2.8–5.6 | 3.2×10^9^ (*A^–^*); 1.3×10^8^ (*HA*)  8.5×10^7^ (*A^–^*); 1.6×10^7^ (*HA*)  7.7×10^6^ (*A^2–^*); 4.7×10^7^ (*HA^–^*); 1.4×10^6^ (*H_2_A*)  5.0×10^8^ (*A^2–^*); 5.0×10^8^ (*HA^–^*); 1.1×10^8^ (*H_2_A*)  3.0×10^8^ (*A^2–^*); 6.0×10^7^ (*HA^–^*); 2.0×10^7^ (*H_2_A*) |  |

Table S3: Second order rate constant with HO^●^ ($k_{HO^{\bullet}} (M^{-1}s^{-1})$) of amino acids and carboxylic acids ^2-4^. For AA the $k_{HO^{\bullet}}$ value is also reported in L molC^-1^ s^-1^ in order to compare the relative contribution of AA reactivity toward HO^●^ in comparison with value estimated for DOC. For AAs the value is given for the deprotonated form while, for CA are given for both deprotonated and protonated forms.

| **Sample** | **DOC (mg C L^-1^)** |  | |
| --- | --- | --- | --- |
|  |  | Without Formate | With Formate |
| 1 | 2.9 | 17.5 ± 3.5 | 0.28 ± 0.06 |
| 2 | 3.4 | 14.5 ± 2.4 | 0.23 ± 0.04 |
| 3 | 6.5 | 23.3 ± 4.1 | 0.37 ± 0.07 |
| 4 | 8.6 | 25.1 ± 4.5 | 0.40 ± 0.07 |
| 5 | 1.5 | 12.1 ± 2.0 | 0.19 ± 0.03 |
| 6 | 1.7 | 14.7 ± 2.6 | 0.24 ± 0.04 |
| 7 | 2.6 | 18.7 ± 3.5 | 0.30 ± 0.06 |
| 8 | 3.9 | 29.8 ± 5.5 | 0.48 ± 0.09 |
| 9 | 4.5 | 31.8 ± 5.7 | 0.51 ± 0.09 |
| 10 | 1.9 | 12.9 ± 2.0 | 0.21 ± 0.03 |
| 11 | 1.8 | 12.5 ± 1.9 | 0.20 ± 0.03 |
| 12 | 1.8 | 16.3 ± 3.1 | 0.26 ± 0.05 |
| 13 | NM | 25.9 ± 4.2 | 0.42 ± 0.07 |
| 14 | 2.3 | 14.4 ± 2.4 | 0.23 ± 0.04 |
| 15 | 2.7 | 11.4 ± 1.9 | 0.18 ± 0.03 |
| 16 | 1.7 | 22.2 ± 3.0 | 0.36 ± 0.05 |
| 17 | 2.2 | 8.3 ± 1.4 | 0.13 ± 0.02 |
| 18 | 2.2 | 7.2 ± 1.3 | 0.12 ± 0.02 |
| 19 | 1.0 | 8.3 ± 1.5 | 0.13 ± 0.02 |
| 20 | 1.2 | 9.5 ± 1.7 | 0.15 ± 0.03 |
| 21 | 1.0 | 9.0 ± 1.7 | 0.14 ± 0.03 |
| 22 | 2.3 | 10.8 ± 2.1 | 0.17 ± 0.03 |
| 23 | 1.8 | 9.1 ± 1.6 | 0.15 ± 0.03 |
| 24 | 1.9 | 11.2 ± 2.1 | 0.18 ± 0.03 |
| 25 | 2.2 | 10.2 ± 1.8 | 0.16 ± 0.03 |

Table S4: DOC concentration for each cloud water sample. are used to determine the HO^●^ scavenging rate competition between amino acids and carboxylic acids. NM: not measured


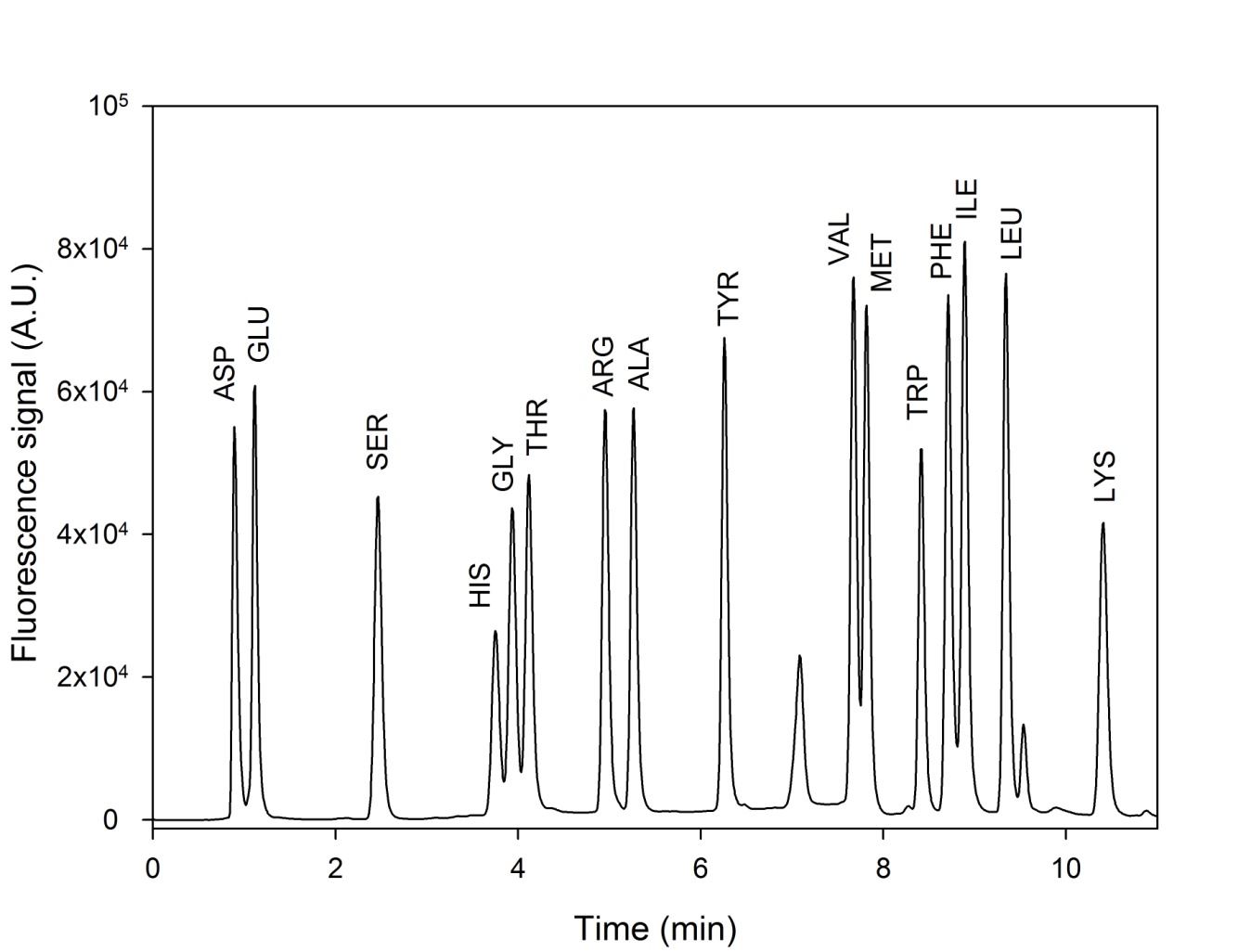


Figure S1: Chromatogram obtained for a standard solution (10 µM of each amino acid; injection volume of 1 µL).

**Determination of contribution of AA to the scavenge of hydroxyl radical *vs* DOC**

Competition between AA and DOC was determined considering the ration between the pseudo-first order scavenge of HO^●^ considering AA and DOC concentrations in each cloud sample ( ).

Data for each cloud sample are summarized in Table S5

| **Sample** | $k_{HO^{\bullet},DOC_{i}}^{'}$(×10^4^) | $\text{ }k_{HO^{\bullet},AA_{i}}^{'}$(×10^4^) |  |
| --- | --- | --- | --- |
|  |  |  |  |
| 1 | 9.18 | 1.52 | 16.5 |
| 2 | 10.8 | 1.26 | 11.7 |
| 3 | 20.6 | 1.72 | 8.4 |
| 4 | 27.2 | 1.89 | 6.9 |
| 5 | 4.75 | 1.05 | 22.1 |
| 6 | 5.38 | 1.28 | 23.7 |
| 7 | 8.23 | 1.62 | 19.7 |
| 8 | 12.4 | 2.19 | 17.7 |
| 9 | 14.3 | 2.28 | 16.0 |
| 10 | 6.02 | 1.12 | 18.6 |
| 11 | 5.70 | 1.08 | 19.0 |
| 12 | 5.70 | 1.42 | 24.9 |
| 13 | 27.2 | 2.25 | 8.2 |
| 14 | 7.28 | 1.25 | 16.4 |
| 15 | 8.55 | 0.99 | 11.1 |
| 16 | 5.38 | 1.93 | 35.8 |
| 17 | 6.97 | 0.72 | 10.3 |
| 18 | 6.97 | 0.63 | 8.3 |
| 19 | 3.17 | 0.72 | 20.7 |
| 20 | 3.80 | 0.82 | 21.6 |
| 21 | 3.17 | 0.78 | 24.6 |
| 22 | 7.28 | 0.94 | 12.9 |
| 23 | 5.70 | 0.79 | 13.8 |
| 24 | 6.02 | 0.97 | 16.1 |
| 25 | 6.97 | 0.88 | 12.7 |

Table S5: scavenge of HO^●^ accounted by for DOC and AA and contribution of amino acids in % for each cloud water sample.

| 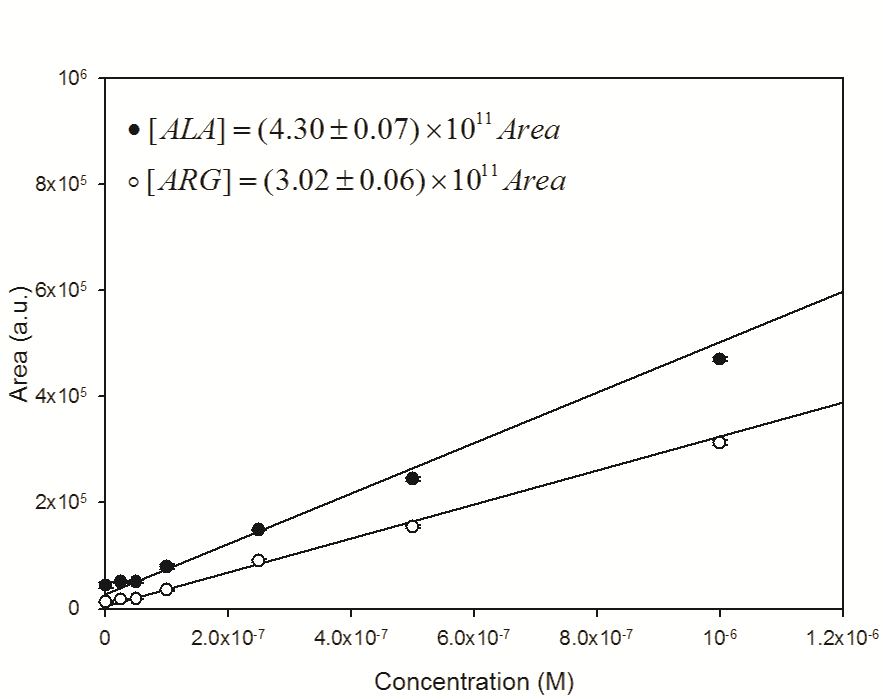 | 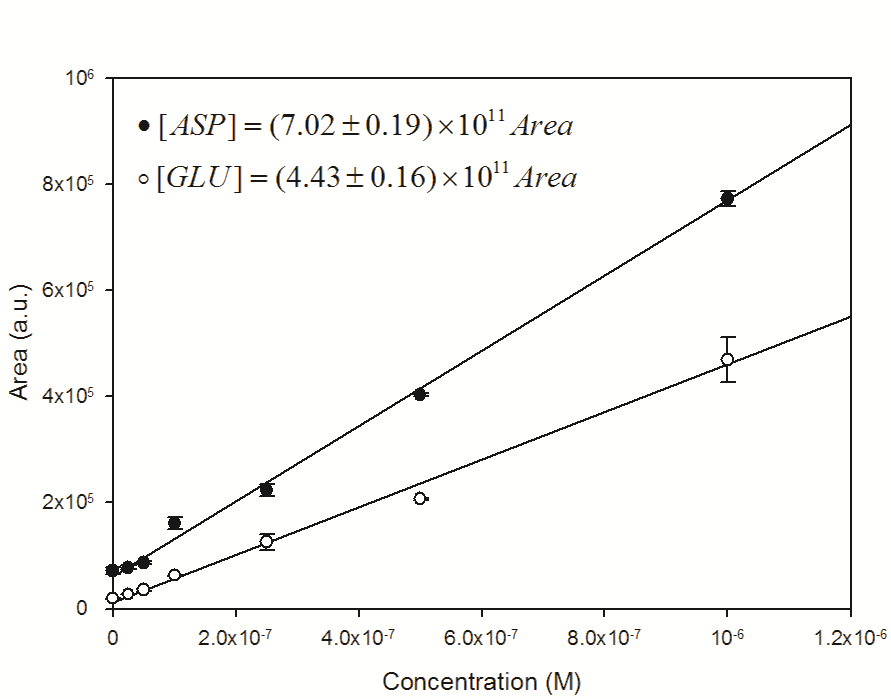 |
| --- | --- |
| 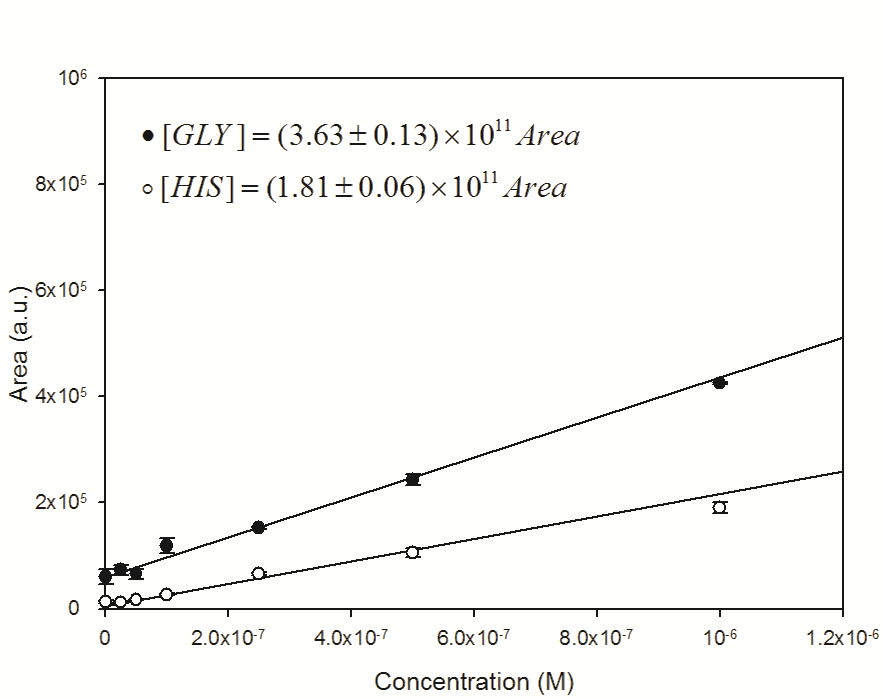 | 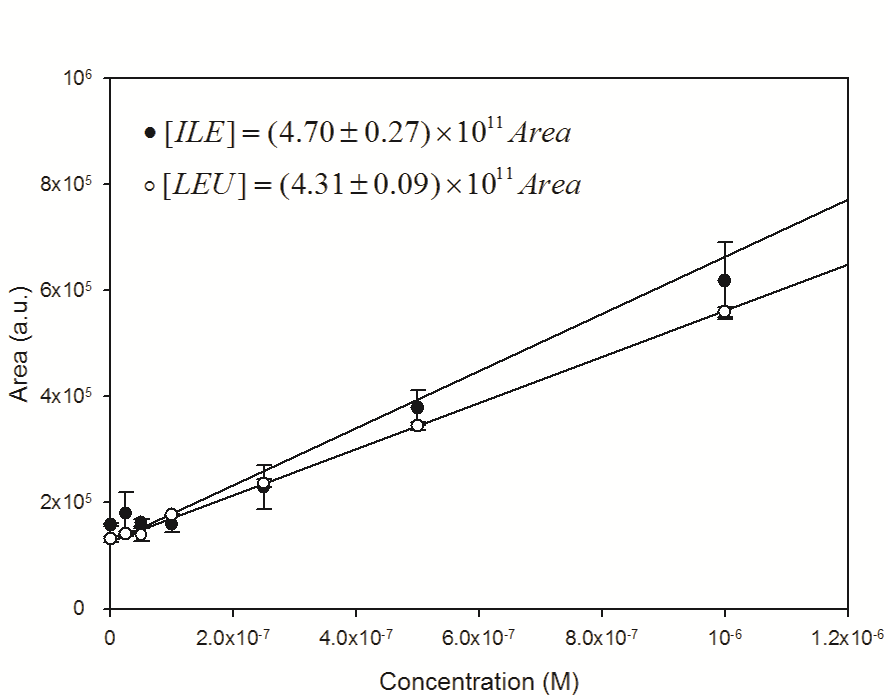 |
| 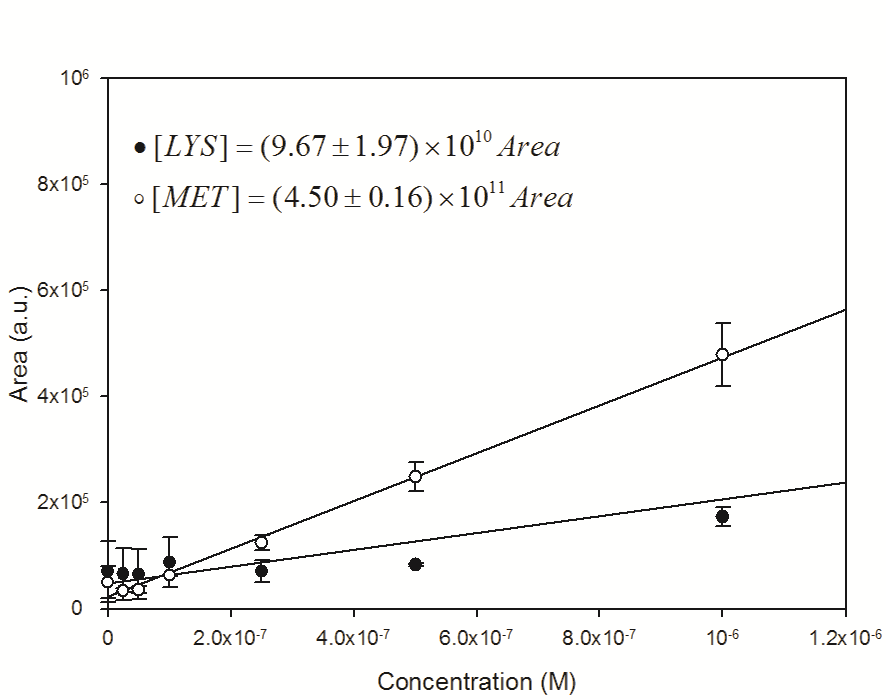 | 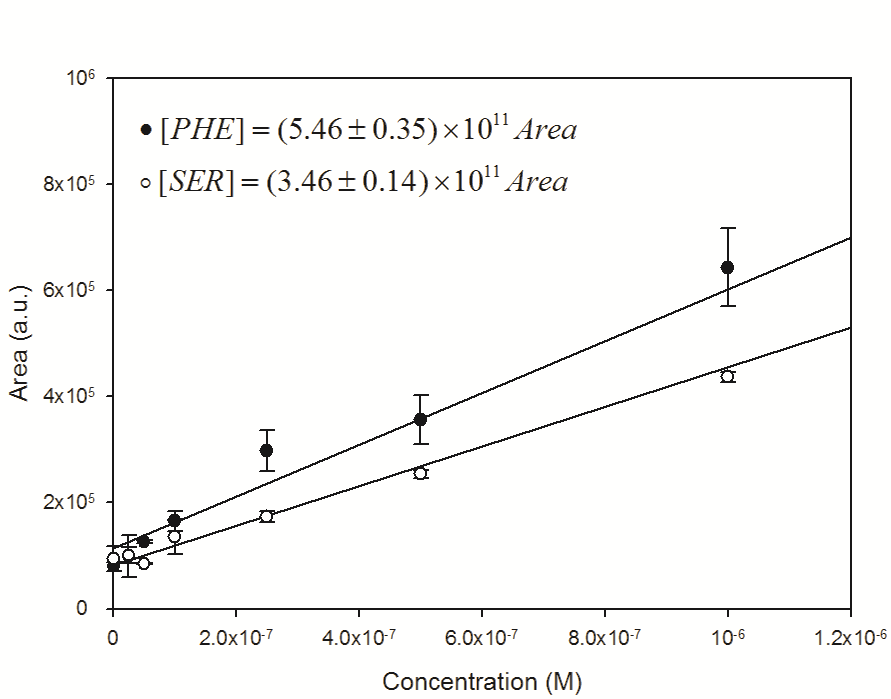 |
| 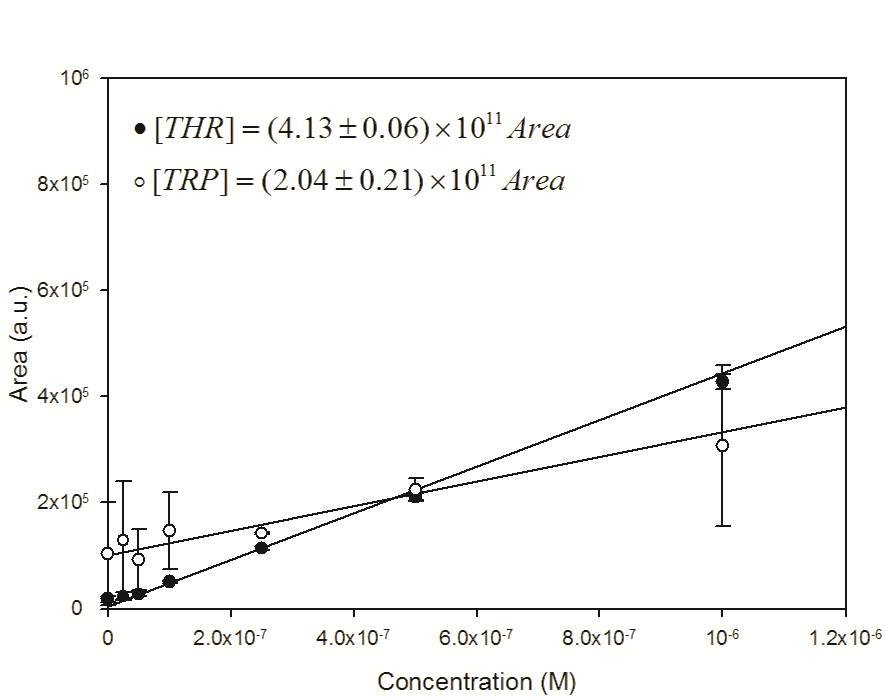 | 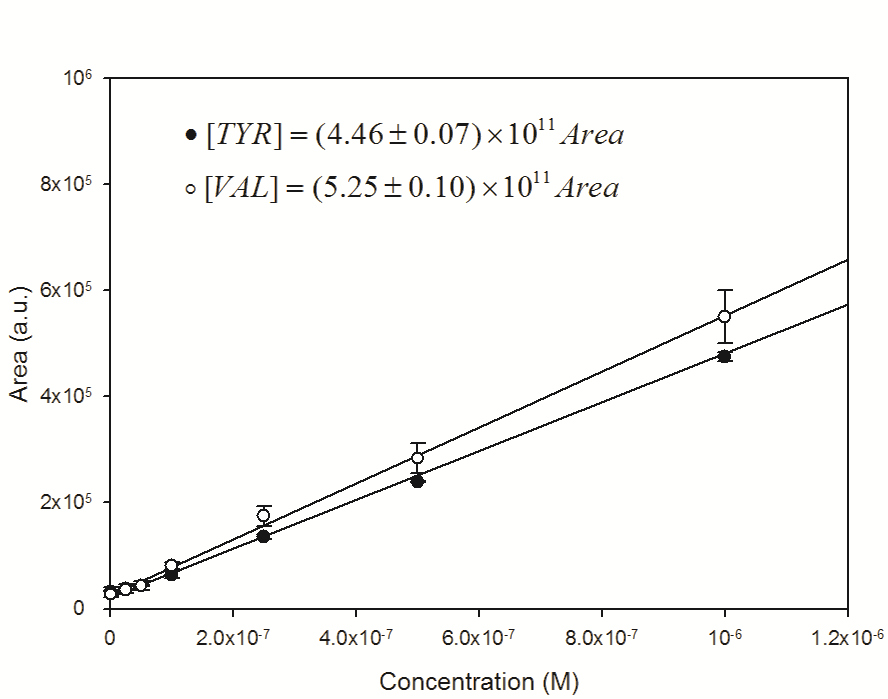 |

Figure S2: Calibration curve of amino acids injection using as matrix a cloud water sample. Calibration curve are obtained with 20 µL volume injection.

References

1. Deguillaume, L. *et al.* Classification of clouds sampled at the puy de Dôme (France) based on 10 yr of monitoring of their physicochemical properties. *Atmos. Chem. Phys.* **14**, 1485-1506 (2014).

2. Buxton, G. V., Greenstock, C. L., Helman, W. P. & Ross, A. B. Critical review of rate constants for reactions of hydrated electrons, hydrogen atoms and hydroxyl radicals (^•^OH/^•^O^−^) in aqueous solution. *J. Phys. Chem. Ref. Data* **17**, 513-886 (1998).

3. Ervens, B., Gligorovski, S. & Herrmann, H. Temperature-dependent rate constants for hydroxyl radical reactions with organic compounds in aqueous solutions. *Phys. Chem. Chem. Phys.* **5**, 1811-1824 (2003).

4. Herrmann, H. Kinetics of aqueous phase reactions relevant for atmospheric chemistry. *Chem. Rev.* **103**, 4691-4716 (2003).
